# The supplementation of dietary black cumin (*Nigella Sativa*) seeds on performance, blood hematology, post-metabolic responses, antioxidant status, immunity, and inflammatory markers in pre-weaning calves

**DOI:** 10.1007/s11250-025-04373-z

**Published:** 2025-04-02

**Authors:** Mamdouh Elsayed, Khaled M. Al-Marakby, Sabry Abdel Hafez, Sameh A. Abdelnour

**Affiliations:** Department of Animal Production, Faculty of Agriculture, Zagazig University, Zagazig, 44511 Egypt

**Keywords:** Performance, Calves, Pre-weaning, *Nigella sativa*, Blood biochemistry, Health

## Abstract

Pre-weaning feeding is critical for calf growth, laying the foundation for future productivity and health. *Nigella sativa* seeds (NS) are rich in bioactive compounds with numerous beneficial effects on health and various pharmacological properties. This study aimed to investigate the supplementation of NS powder on performance, post-metabolic attributes, immunity, antioxidant capacity, and inflammatory responses in pre-weaning Friesian calves. Twenty-four Friesian male calves at 4 days of age with a similar genetic line, weighing 33.67 ± 0.6 kg, were randomly allocated to three groups (8 animals per group). The study comprised three groups: a control group (NS0) receiving no supplementation, and two experimental groups received either 1% (NS1) or 3% (NS3) NS supplementation for 84 days. All levels of NS supplementation significantly improved the final body weight and body weight gain in a linear manner (*P* < 0.001), while the highest total dry matter intake was observed in NS1 group (quadratic; *P* < 0.001). White blood cells (quadratic, *P* = 0.026), lymphocytes (quadratic, *P* = 0.012), and monocytes (linear effect; *P* = 0.001) significantly decreased, whereas red blood cells (linear; *P* = 0.004), hematocrit (linear; *P* = 0.002), mean corpuscular volume (MCV, linear; *P* = 0.003), and mean corpuscular hemoglobin concentration (MCHC, quadratic, *P* = 0.007), platelets (linear; *P* < 0.001) increased in calves fed NS. Feeding calves diets supplemented with NS led to a significant linear decrease in plasma creatinine and liver enzymes (AST and ALT) compared to the control diet (*P* < 0.01). Calves fed 3% of NS in their diets had lower plasma cholesterol (linear; *P* < 0.001) and triglyceride levels (linear; *P* = 0.002) compared to calves in NS0 and NS1 groups. Polynomial analysis indicated a quadratic decrease in direct bilirubin (*P* = 0.006), and a quadratic increase in immunoglobulin G (IgG, *P* = 0.014) and M (IgM, *P* = 0.032) in the calves fed the NS diet. All NS-supplemented groups showed a significant increase in IL-10 (linear; *P* < 0.001), TAC (linear; *P* = 0.006), and CAT (linear; *P* < 0.001), and a significant decrease in IL-4 levels (linear; *P* < 0.001) of the plasma of pre-weaning calves. As expected, pre-weaning calves fed diets containing NS (1% or 3%) exhibited a quadratic decrease in plasma malondialdehyde (MDA, *P* < 0.001) levels compared to those fed diets without NS. Our findings suggest that incorporating up to 3% *Nigella sativa* into pre-weaning calf diets can enhance growth, bolster immune function, and mitigate oxidative stress, offering a promising strategy for improving health and sustainability on dairy farms.

## Introduction

Pre-weaned calves are particularly vulnerable to environmental, managerial, and disease-related challenges, leading to increased mortality rates during their early life (Lorenz et al. 2011). Their underdeveloped immune systems, combined with unsuitable environmental factors and diseases, make them prone to respiratory and intestinal infections, potentially causing growth retardation and compromised health (Savvidou et al. 2023). Although antibiotics have been employed to reduce diarrhea-related morbidity and mortality, stricter regulations in the livestock industry, such as the EU ban on antibiotics and ionophores and the US Veterinary Feed Directive, have necessitated a shift towards alternative strategies (Hayajneh et al. 2024). Consequently, natural feed additives, such as medicinal plants (Batool et al. 2023; Khan et al. 2023), have gained increasing attention in recent decades as a scientific approach to promoting optimal growth, development, and health in early life ( Wang et al. 2024; Rashid et al. 2024). These innovative interventions offer significant promise for the future of the livestock industry.

Black cumin (*Nigella sativa*), an annual herb from the Ranunculaceae family, is widely cultivated, especially in the Middle East (Obeidat and Alqudah 2023). Black cumin seeds offer a potential alternative feed source. This versatile plant is used both as a food additive and medicinal plant and is rich in various bioactive compounds and essential nutrients (Ciesielska-Figlon et al. 2023) containing thymoquinone (TQ), which is believed to be responsible for many of its therapeutic effects (Dabeer et al. 2022). Its seeds are a nutritional powerhouse, rich in proteins (20.0–27.0%), carbohydrates (23.50–33.20%), fats (34.5–38.7%), fiber (8.4%), and minerals, as well as vitamins and carotene (Dabeer et al. 2022).

Beyond their nutritional value, black seeds contain bioactive compounds like thymohydroquinone, TQ, and nogelleone, which contribute to their antioxidant, antimicrobial, and immune-boosting properties (Lorenz et al. 2011; Majeed et al. 2021). These properties have made NS and their extracts popular in traditional medicine for various ailments (Shahzad et al. 2009). Given their potent antioxidant and immune-stimulating effects (Ciesielska-Figlon et al. 2023; Meddah et al. 2024), black seeds may offer potential benefits in mitigating stress induced by high temperatures and humidity. Furthermore, NS enriched with nano-selenium exhibits antimicrobial activity against *Salmonella* spp. (Abdalhamed et al. 2023), a major cause of infectious diarrhea in ruminants. Studies in various animal species have demonstrated the beneficial effects of NS on growth, milk production (Obeidat and Alqudah 2023) and reproductive efficiency (Selim et al. 2019; Mohammed and Al-Suwaiegh 2023; Fathi et al. 2024). These studies suggest that dietary NS can positively impact growth parameters, milk yield, plasma metabolites, and reproductive efficiency. However, few studies have investigated the use of NS to improve the health and growth of pre-weaning calves.

Given the diverse biological activities reported for NS seeds and its derivatives, we hypothesized that NS supplementation would improve immunity and antioxidant status, mitigate oxidative stress and inflammation, and ultimately promote growth during the pre-weaning period in calves. There is limited research on the effects of *N. sativa* in the starter diets of pre-weaned calves, particularly regarding their impact on growth, post-metabolic responses, feed efficiency, immunity, redox status, and inflammatory responses. Therefore, our hypothesis is that supplementing the diet of pre-weaned calves with *N. sativa* seeds will improve performance, enhance overall health, and reduce the risks associated with the pre-weaning period.

## Material and methods

### Location of study and ethical statement

This study was conducted at a private dairy farm, Ismailia-Cario Road, about 70 km to Zagazig. All procedures and experimental protocols adhered to Directive 2010/63/EU of the European Parliament and the Council of 22 September 2010 legislation on the protection of animals used for scientific purposes. The experimental procedures were approved by the Zagazig University Scientific Research Ethics Committee (Ethical code: ZU-IACUC/2/F/174/2022) and conducted in accordance with the ARRIVE guidelines 2.0.

### *Nigella sativa* source

Black cumin (*Nigella sativa)* seeds were purchased from Ab Chem Company for Chemical Raw Materials, Mansoura, Egypt. The seeds were authenticated by Professor El-Sayed M. Desoky, Plant Department, Faculty of Agriculture, Zagazig University. The seeds were ground to a fine size (0.2 mm) and stored in the dark until they were incorporated into the diets according to the study protocol.

### Experimental animals and diets

A total of twenty-four male Friesian calves at 4 d of age with a similar genetic line under a completely randomized design were used in this study. The animals were assigned into three various treatment groups, comprising of eight calves in each. The average weight of the calves was 33.67 ± 0.6 kg at the start of the experiment. Directly after birth, the calves received 10% colostrum milk based on body weight at birth, followed by transition milk until the third day of life. Then, calves were transferred to the trial facility where they were kept for a duration of 12 weeks.

The calves received milk plus a starter mixture served as the control group (free NS; NS0 group), while the starter mixture was reinforced with 1% and 3% of NS as NS1 and NS3 groups, respectively, for 84 days. The tested NS dosage was preferred based on the results of El-Nagar et al. (2023). Milk was provided as 15, 10, and 5% of live body weight per day during the periods 4–60, 61–74, and 75–88 days of age, respectively. Calves were housed in individual pens (1.5 m length × 1.0 m width) with rice straw as the bedding material throughout the trial. Feed and water were provided *ad libitum*. The starter feed was prepared according to the guidelines of Drackley (2008).

The ingredients and chemical composition of the starter mixture are shown in Table 1. The chemical composition of the starter mixture was determined using standard AOAC methods (AOAC 1995). These methods were used to analyze the levels of crude ash (954.01), dry matter (925.10), ether extract (920.39), crude fiber (920.85), and crude protein (Kjeldahl method, 954.01). All laboratory analyses were conducted on a dry matter basis.

**Table 1:** Ingredients chemical composition of the starter mixture fed to calves

Item	Experimental diets		
	Control	NS1	NS2
Yellow corn	350	350	350
Alfalfa hay	150	150	150
Wheat bran	95	90	80
Barley grain	100	95	85
Soybean meal, 44% CP	250	250	250
Molasses	30	30	30
Iodized sodium chloride	8	8	8
Calcium phosphate	10	10	10
Mineral and vitamin premix ^1^	7	7	7
Black seed	0	10	30
Total	1000	1000	1000
**Chemical analysis (% on dry matter basis)**			
DM	88.65	88.64	89.19
Ash	8.52	8.37	8.04
OM	91.48	90.03	90.6
EE	1.93	2.09	2.25
CF	9.94	9.82	9.61
CP	21.19	21.29	21.49
NFE	58.42	56.83	57.25

^1^ Each kilogram of premix contains: vitamin A, 50,000 IU; vitamin D3, 10,000 IU; vitamin E, 2900 IU; Ca, 196 g; P, 96 g; MgSO_4_, 19,000 mg; FeSO_4_, 9000 mg; CuSO_4_, 5000 mg; Mn, 6000 mg; ZnSO_4_, 8000 mg; Co, 20 mg; I, 227 mg; Se, 67 mg. Dry matter (DM), organic matter (OM), Ether extract (EE), Crude fibre (CF), Crude protein (CP), and nitrogen free extract (NFE)

### Performance traits

The weights of calves in this experiment were weighted using a digital scale (Tru-Test 702, Tru-Test Datamars, Lugano, Switzerland). The body weight was assessed every two weeks to adjust milk and feed requirements. However, in this study, the final body weight was used as the growth index.

The average total milk intake (TMI) and total dry matter intake (TDMI; from milk and starter) were calculated over the entire period. Body weight gain was determined by subtracting the initial body weight (IBW) from the final body weight (FBW).

$$\mathrm{WG}\;(\mathrm{weight}\;\mathrm{gain})=\mathrm{FBW}\;(\mathrm{final}\;\mathrm{body}\;\mathrm{weight})-\mathrm{IBW}\;(\mathrm{initial}\;\mathrm{body}\;\mathrm{weight}).$$

$$\mathrm{TDMI}=(\mathrm{Dry}\;\mathrm{matter}\;\mathrm{from}\;\mathrm{milk})+(\mathrm{Dry}\;\mathrm{matter}\;\mathrm{from}\;\mathrm{starter}).$$

The average daily gain (ADG) was calculated by dividing the difference between FBW and IBW by the number of days. The feed conversion ratio (FCR) was calculated by dividing the average TDMI by the ADG. FCR (Feed intake/ weight gain).

### Blood samples

Blood samples at 88 days of age were taken from the jugular vein 3 h ± 30 min after feeding the calves in the morning. Blood samples were collected into tubes with an anticoagulant (K3-EDTA) for hematological profile and plasma collection to evaluate post-metabolic, immunity, antioxidant and inflammatory status. The samples were centrifuged at 503 × *g* at 4 °C for 15 min. The supernatant was then collected into Eppendorf tubes (1.5mL) and stored in a deep freezer at − 20 °C for subsequent plasma attributes. The hematology reports were estimated utilizing an automated hematology analyzer (Medonic CA620 VET, Stockholm, Sweden). The following blood attributes were assessed: hemoglobin (HGB) level, red blood cell (RBCs) count, hematocrit (HCT) value, mean corpuscular hemoglobin concentration (MCHC), mean corpuscular volume (MCV), platelet count (PLT), mean corpuscular hemoglobin (MCH), white blood cell (WBCs) count, and the percentages of lymphocytes (LYM) and monocytes (MON).

### Post-metabolic response

The contents of total cholesterol (TC), aspartate (AST), and alanine (ALT) transaminases, triglyceride (TG), urea, high-density lipoprotein (HDL), creatinine, total, direct bilirubin and indirect bilirubin, very low-density lipoprotein (VLDL), and low-density lipoprotein (LDL) were assessed from the plasma samples using commercial kits (Diamond Diagnostic, Dokki, Giza, Egypt) following the manufacturer’s instructions. Glucose was measured using the Glucose Oxidase Assay Kit (code: K-GLOX) provided by Megazyme Company (Bray Business Park, A98 YV29 Bray, Ireland).

### Immunoglobins determination

The levels of plasma IgG (SKU: BGG69-K01) and IgM (SKU: BCM61-K01) were evaluated using the enzyme-linked immunosorbent assay (ELISA) with a bovine commercial kit provided by Eagle Biosciences (State Route 101A, Unit 12, Amherst, USA) according to the manufacturer's instructions. The sensitivity values were 3.439 ng/mL and 3.006 ng/mL, and the dynamic range values were 7.81–500 ng/mL and 12.5–400 ng/mL for IgG and IgM, respectively.

### Pro-inflammatory response

The levels of interleukin-4 (IL-4, Code: CSB-E12898B), and interleukin-10 (IL-10, Code: CSB-E12917B) were measured in the plasma using a colorimetric ELISA Sandwich method according to the manufacturer's instructions (CUSABIO, Wuhan, Hubei Province 430,071, China). The detection values were 12.5 −800 pg/mL and 5 −1000 pg/mL, while the sensitivity values were 3.12 pg/mL- 2.5 pg/mL for IL-4 and IL-10.

### Redox status determination

The levels of catalase (CAT), superoxide dismutase (SOD), and total antioxidant capacity (TAC) were assessed using various methods Szymonik-Lesiuk et al. (2003), Marklund et al. (1982), and Prior and Cao (1999), respectively with BioMérieux's (Marcyl'Etoile, France) commercial kits following the manufacturer's guidelines. Oxidative-related biomarkers such as lipid peroxidation (MDA) were assessed according to the method proposed by Tsikas (2017) using a commercial kit purchased from Abcam.

#### Data analysis

The normality test and homogeneity of variance were evaluated using Shapiro–Wilk and Levene's tests, respectively. Orthogonal contrast statements were used to assess linear and quadratic dose–response relationships for each dependent variable across different NS levels (0, 1%, and 3% of the diet). Duncan's multiple range test was applied for post-hoc comparisons. Statistical analyses were performed using SPSS 25.0 (SPSS Inc., Chicago, IL, USA) with a mixed-effects model (PROC MIXED). The significance level was set at a P ≤ 0.05.

## Results

### Impacts on growth indices

**Fig. 1:**
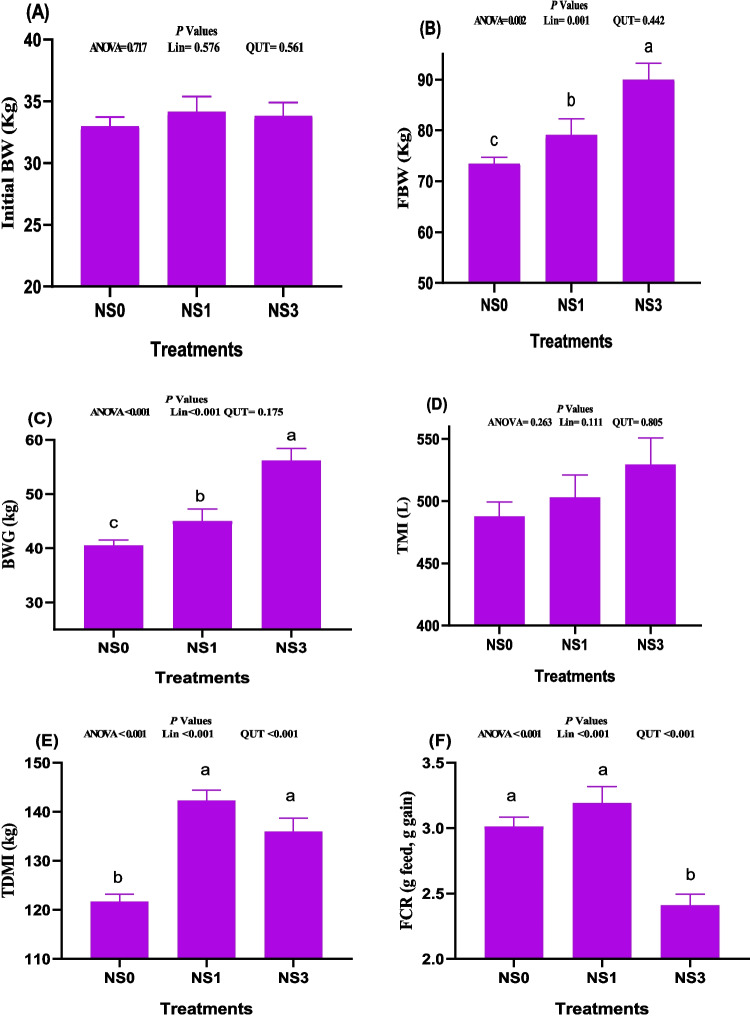
A-E. The impacts of dietary administration of *Nigella sativa* on growth attributes such as initial body weight (Fig. 1A), final body weight (FBW, Fig. 1B), body weight gain (BWG, Fig. 1C), total milk intake (TMI, Fig. 1D), total dry matter intake (TDMI, Fig. 1E) and feed conversion ratio (Fig. 1F) of growing calves after 3 months of feeding. ^2^NS0, NS1 and NS3; calves were fed diets supplemented with 0, 1 or 3% of *Nigella sativa*. *P* value was considered at *P* < 0.05 using polynomial analysis; LIN = linearity, and QUT = quadratic. Data are presented as means ± SE

The impacts of the various levels of NS on the growth performance of pre-weaning calves are shown in Fig. 1(A-F). The initial body weights (IBW, *P* > 0.05) were similar in all experimental groups (Fig. 1A). The FBW (Fig. 1B) and BWG (Fig. 1C) were significantly the highest and lowest in NS3 and NS0, respectively (*P* < 0.05). Additionally, the average values of TMI (Fig. 1D) were slightly better (P > 0.5) in NS1 (the best) and NS3 compared to the control one. The dietary NS improved the TDMI (Fig. 1E) compared to the control. Where the highest TDMI (Fig. 1E) was observed in pre-weaning calves that fed 1% NS in their diet (quadratic; *P* < 0.001). Intermediate values of TDMI were observed in the NS3 group (*P* < 0.001). Hence, the best value of FCR (Fig. 1F) was observed with the highest NS level (NS3).

### Impacts on blood hematology

**Fig. 2:**
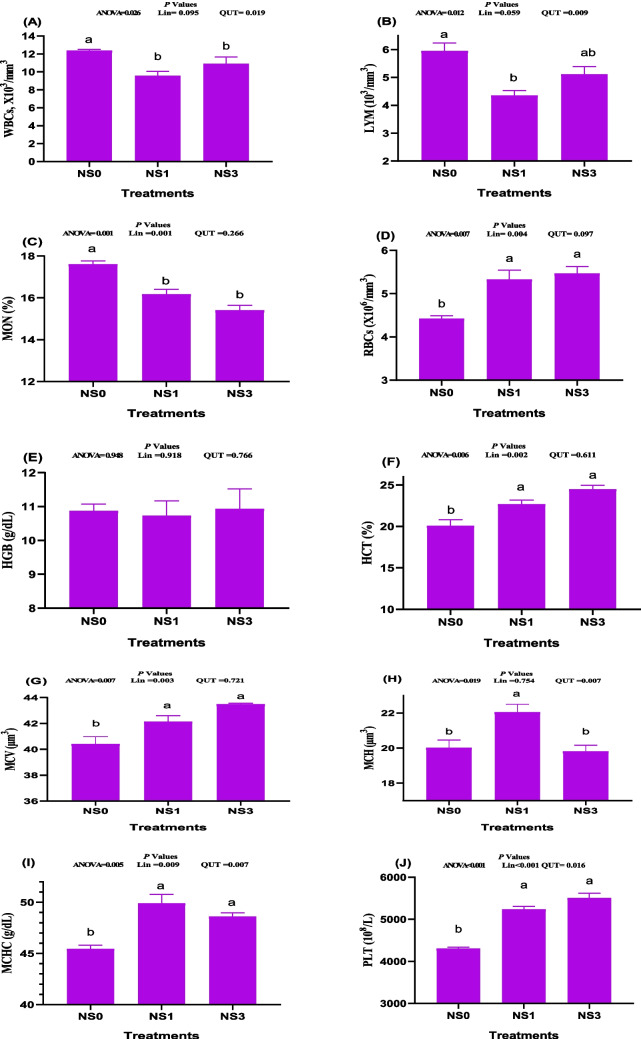
A-J. The impacts of dietary administration of *Nigella sativa* on the blood hematological attributes including white blood cells (WBCs, Fig. 2A), lymphocytes (LYM, Fig. 2B), monocytes (MON, Fig. 2C), red blood cells (RBCs, Fig. 2D), hemoglobin (HGB, Fig. 2E), hematocrit (HTC, Fig. 2F), mean corpuscular volume (MCV, Fig. 2G), mean corpuscular hemoglobin (MCH, Fig. 2H), Mean corpuscular hemoglobin concentration (MCHC, Fig. 2I), and platelets (PLT, Fig. 2J) of growing calves after 3 months of feeding

According to the data in Fig. 2, there were significant effects of dietary NS addition on the blood hematology of pre-weaning calves (*P* < 0.05). The values of WBCs (*P* = 0.026, Fig. 2A), LYM (*P* = 0.012, Fig. 2B), and MON (linear effect; *P* = 0.001, Fig. 2C) were significantly decreased in all calves received dietary NS. Feeding calves with NS led to a significant increase in RBCs (linear; *P* = 0.004, Fig. 2D), HCT (linear; *P* = 0.002, Fig. 2F), MCV (linear; *P* = 0.003, Fig. 2G), MCHC (quadratic, *P* = 0.007, Fig. 2I), and platelets (linear; *P* < 0.001, Fig. 2J) compared to the NS0 group. HGB (Fig. 2E) did not differ among all groups (*P* > 0.05). The NS1 group exhibited a significantly higher MCH compared to the other groups, following a quadratic trend (*P* = 0.007, Fig. 2H).

### Impacts on post-metabolic response

Plasma glucose and urea levels were not affected by the NS addition (*P* > 0.05; Table 2). Diets involving NS resulted in a significant linear reduction in plasma creatinine concentrations (*P* < 0.01), and linear insignificant decreases (*P* > 0.05) in urea levels compared to the control diet. Compared to the NS0 and NS1 groups, calves fed 3% NS had significantly lower plasma cholesterol (linear; *P* < 0.001), LDL (*P* = 0.03), and triglyceride (linear, *P* = 0.002) levels. While HDL and VLDL levels were similar across all groups, a non-significant trend towards lower levels was observed in the NS3 group (*P* > 0.05). All NS-supplemented groups showed significant reductions in liver enzymes AST (linear; *P* = 0.002) and ALT (linear; *P* = 0.001), with the lowest values observed in the NS3 group. Total and indirect bilirubin levels did not differ significantly among the groups (*P* > 0.05). Whereas the direct bilirubin levels decreased quadratically with NS supplementation (*P* = 0.006). Overall, dietary NS supplementation appears to improve liver health, kidney function, and potentially reduce the lipid profile in pre-weaning calves.

**Table 2:** The impacts of dietary administration of *Nigella sativa* on the blood biochemistry attributes of growing calves after 3 months of feeding

Item^1^		Experimental groups^2^			*P* values^3^		
		NS0	NS1	NS3	ANOVA	LIN	QUT
Glucose	mg/dL	77.00 ± 2.65	77.33 ± 0.88	76.00 ± 0.58	0.841	0.682	0.693
**Kidney function**							
Creatinine	g/dL	1.23^a^ ± 0.03	1.08^b^ ± 0.03	1.01^b^ ± 0.02	0.002	0.001	0.211
Urea	g/dL	31.42 ± 1.18	30.44 ± 1.37	27.97 ± 0.62	0.154	0.069	0.599
**Lipid profile**							
Cholesterol	mg/dL	175.67^a^ ± 2.7	174.33^a^ ± 3.7	134.00^b^ ± 1.53	< 0.001	< 0.001	0.001
Triglycerides	mg/dL	106.33^a^ ± 3.7	101.33^a^ ± 1.2	87.67^b^ ± 2.33	0.006	0.002	0.226
HDL	mg/dL	47.96 ± 1.02	49.73 ± 1.63	49.22 ± 0.97	0.611	0.502	0.482
LDL	mg/dL	97.73^b^ ± 2.4	108.55^a^ ± 4.9	92.38^c^ ± 1.2	0.030	0.282	0.014
VLDL	mg/dL	20.64 ± 0.60	21.29 ± 0.74	18.99 ± 0.99	0.188	0.191	0.180
**Liver function**							
ALT	IU/L	26.33^a^ ± 1.45	23.67^b^ ± 0.67	17.33^c^ ± 1.20	0.004	0.001	0.242
AST	IU/L	28.33^a^ ± 0.88	26.00^b^ ± 0.58	23.00^c^ ± 0.58	0.005	0.002	0.708
Total bilirubin	mg/dL	0.96 ± 0.11	0.87 ± 0.06	0.88 ± 0.02	0.640	0.446	0.605
Direct bilirubin	mg/dL	0.27^a^ ± 0.01	0.20^b^ ± 0.01	0.22^b^ ± 0.01	0.004	0.007	0.006
Indirect bilirubin	mg/dL	0.66 ± 0.06	0.67 ± 0.06	0.66 ± 0.02	0.987	1.000	0.875

^1^ High-density lipoprotein (HDL), low-density lipoprotein (LDL), very high-density lipoprotein (VLDL), alanine aminotransferase (ALT), Aspartate aminotransferase (AST)

^2^NS0, NS1 and NS3, calves were fed diets supplemented with 0, 1 or 3% of *Nigella sativa*

^3^*P* value was considered at *P* < 0.05 using polynomial analysis; LIN = linearity, and QUT = quadratic. Data are presented as means ± SE

### Impacts on immunity, redox state, and inflammatory response

In comparison with the NS0, polynomial analysis revealed a quadratic increase in plasma IgG (*P* = 0.014) and IgM (*P* = 0.032) levels in pre-weaning calves fed diets containing NS (Table 3). NS-supplementation significantly decreased the plasma levels of IL-4 (*P* < 0.001) and MDA (*P* < 0.001), however increased the levels of IL-10 (linear; *P* < 0.001) compared to the control group. The levels of TAC (linear; *P* = 0.006) and CAT (linear; *P* < 0.001) were also significantly higher in the NS groups compared to the control group.

**Table 3:** The impacts of dietary administration of *Nigella sativa* on the immune response, inflammation and redox status of growing calves after 3 months of feeding

Item^1^		Experimental groups^2^			*P* values^3^		
		NS0	NS1	NS3	ANOVA	LIN	QUT
Immune response							
IgM	mg/L	21.60^b^ ± 0.40	25.60^a^ ± 0.75	24.83^a^ ± 0.87	0.015	0.017	0.032
IgG	mg/L	41.88^b^ ± 0.72	49.88^a^ ± 1.45	49.27^a^ ± 0.74	0.003	0.002	0.014
Inflammatory response							
IL-4	ng/mL	72.13^a^ ± 1.02	45.34^b^ ± 1.34	45.01^b^ ± 2.21	< 0.001	< 0.001	0.001
IL-10	ng/mL	84.65^b^ ± 3.85	121.61^a^ ± 3.89	126.90^a^ ± 2.63	< 0.001	< 0.001	0.010
Redox status							
TAC	mmol/L	2.80^b^ ± 0.09	3.45^a^ ± 0.18	3.52^a^ ± 0.07	0.010	0.006	0.097
CAT	mmol/L	10.16^b^ ± 0.53	18.65^a^ ± 0.85	18.79^a^ ± 1.15	0.001	< 0.001	0.008
MDA	mmol/L	233.00^a^ ± 5.29	104.07^b^ ± 1.67	106.07^b^ ± 2.72	< 0.001	< 0.001	< 0.001

^1^Immunoglobulins M(IgM), G (IgG), interleukin-4 (IL-4), interleukin-6 (IL-6), total antioxidant capacity (TAC), catalase (CAT), malondialdehyde (MDA)

^2^ NS0, NS1 and NS3, calves were fed diets supplemented with 0, 1 or 3% of *Nigella sativa*

^3^*P* value was considered at *P* < 0.05 using polynomial analysis; LIN = linearity, and QUT = quadratic. Data are presented as means ± SE

## Discussion

The pre-weaning period is critical for calves due to their increased susceptibility to diarrhea and gastrointestinal disease, leading to an increase in mortality rates. Immunomodulatory agents have emerged as a promising strategy to bolster calf health and mitigate these risks, ultimately improving economic returns for dairy farms. *Nigella sativa,* a widely studied herb renowned for its diverse health benefits and pharmacological properties, was investigated in this study. Specifically, we examined how dietary *N. sativa* supplementation impacts blood health, growth, antioxidant status, and immune function in pre-weaning calves. Our results demonstrate that supplementing starter diets with 1% or 3% *N. sativa* seeds promoted growth, augmented immunity, enhanced health and reduced oxidative stress and inflammation in pre-weaning calves. These findings underscore the potential of natural compounds like *N. sativa* to support sustainable dairy farming practices.

Growth performance is a crucial metric for evaluating novel feed additives and directly impacts farm profitability. Natural herbs with antioxidants, immunomodulatory, and anti-inflammatory properties have been widely used to enhance growth and animal health. *Nigella sativa* (NS) contains several bioactive compounds, including TQ, P-cymene, *α*-thujene, *γ*-terpinene, carvacrol, *α*-pinene, limonene, and *β*-pinene, which contribute to its diverse health benefits.

This study found that adding 1% or 3% NS to the starter diet positively affected growth, which aligns with the findings of Khalaf et al. (2024), who also demonstrated that the supplementation of 3% NS in broiler diet improved growth performance traits. Similarly, El-Nagar et al. (2023) reported that the supplementation of 1% NS oil in the starter diet of calves resulted in a significant increase in live body weight. Supplementing NS in the diets of various farm animals, including buffalo (Khattab et al. 2011), calves (Abdel-Magid et al. 2007; El-Nagar et al. 2023), goats (Zaher et al. 2020) and lambs (Taha 2017; Retnani et al. 2019; Obeidat 2020), have been identified to improved growth performance traits. The higher performance traits in calves supplemented with NS could be associated with higher amount of protein, minerals, fatty acids and essential amino acid in NS, increasing the availability of nutrients for growth and development and repair of worn-out tissues. Additionally, it could be possible that NS supplementation stimulated the secretion of digestive enzymes, thereby improving nutrient digestion (El-Nagar et al. 2023). Additionally, Abd El-Hafeez et al. (2014) previously reported the absence of diarrhea symptoms in calves supplemented with NS. The growth-promoting effects of black seed may be attributed to its high content of fatty acids and essential amino acids. Furthermore, NS has been shown to enhance digestive enzyme activity and stimulate gastrointestinal motility, leading to improved feed utilization and conversion (Wei et al. 2022). The authors also proposed that the observed improvements could be attributed to the antimicrobial (Randhawa et al. 2017) and antidiarrheal (Safitri et al. 2025) properties of NS.

Blood hematology provides valuable insights into an animal's health status, reflecting the influence of environmental, nutritional, and physiological factors. In this study, NS supplementation improved HTC, RBCs, MCH, MCHC, and PLT (*P* < 0.05) and HGB (*P* > 0.05) values compared to the NS-free diet. These findings align with previous results of Abd El-Hafeez et al. (2014), who reported significant improvements of HGB and HTC in calves fed NS compared to control. Similarly, El-Nagar et al. (2023) observed increased RBC count in Friesian calves supplemented with NS oil. Several studies align with the present results, demonstrating a significant increase in hematological parameters of RBCs, HGB, and PCV in lambs (Al-dain and Jarjeis 2015) and goats (Zaher et al. 2020) fed diets containing NS. Our findings are consistent with those of Khattab et al. (2011), who reported improved hematological parameters in NS oil-treated calves. The observed decrease in WBCs and its fractions (LYM and MON) in our treatment groups may suggest a reduction in microbial infection.

Blood biochemistry reflects the post-metabolic pathways to nutrient absorption and metabolism. Feeding calves a diet containing 3% NS resulted in a considerable reduction in the lipid profile, specifically cholesterol and triglycerides, while significantly improving LDL levels. Furthermore, all NS groups exhibited lower levels of AST, ALT, creatinine direct bilirubin (*P* < 0.05) and urea and total bilirubin (*P* > 0.05) compared to the control diet. These findings are consistent with those of El-Nagar et al. (2023), who reported significant reductions in AST, ALT, urea, and creatinine concentrations in calves fed NS in their starter diets. NS has demonstrated nephroprotective effects by reducing blood urea and creatinine levels in lambs (Al-dain and Jarjeis 2015). This aligns with the findings of (Zaher et al. 2020) who reported significantly lower serum ALT activity in goats supplemented with NS. However, these results contrast with those of El-Nagar et al. (2023), who found that NS increased total lipids and triglycerides in the serum of calves fed NS. These findings suggest that NS possesses hepato-renal protective effects. Authors hypothesized that this protective effect may be attributed to the presence of TQ in NS. Essential oils have a hypocholesterolemic effect by inhibiting 3-hydroxy-3-methylglutaryl coenzyme A reductase, a key enzyme in the regulation of the cholesterol synthesis pathway, due to their active phenolic contents (Weerawatanakorn et al. 2024). In contrast, Abd El-Hafeez et al. (2014) found that dietary inclusion of NS (0.06 mL/g BW) significantly reduced serum glucose and total lipids, our study detected no change in glucose levels with NS administration.

Homeostasis is the tendency of an organism to maintain a stable internal state. During the growth of calves in the suckling period, they are exposed to various environmental issues that can disrupt the balance between oxidative and antioxidant statuses in cellular systems. Calves need to support their health in the first days of life to counteract and support the body's antioxidative status. Some scientists suggest that providing dairy with natural antioxidants may be beneficial to support the cellular antioxidant state and reduce oxidative damage in body tissues. NS is popular for its antioxidant capacity, especially due to its richness in vitamins, minerals, and phenolic compounds such as TQ, P-cymene, *α*-thujene, *γ*-terpinene, carvacrol, *α*-pinene, limonene, and *β*-pinene. The present study indicates that the values of TAC (linear; *P* = 0.006) and CAT (linear; *P* < 0.001) were significantly higher in pre-weaning calves fed NS at 1% or 3% in their diets compared to the control group. As expected, feeding pre-weaning calves diets with NS (1% or 3%) quadratically reduced the MDA levels in their plasma compared to the free-NS diets.

Catalase is a unique antioxidant enzyme due to its rigid structure. It plays a crucial role in the body by breaking down hydrogen peroxide (H_2_O_2_) into water and oxygen. Catalase works in conjunction with superoxide dismutase (SOD), which produces H_2_O_2_ from superoxide. Without catalase, excess H_2_O_2_ generated by SOD can react with metal ions, leading to the formation of the highly destructive hydroxyl radical. The TQ in NS can support the production of catalase in the body, as shown in a study by (Fathi et al. 2024). They reported that the dietary inclusion of NS (up to 15 g/kg diet) significantly improved Catalase and total antioxidant capacity (TAC) in broilers exposed to heat stress. The NS oil, rich in the antioxidant nigellone, may play a crucial role in modulating the initial stages of the bovine calf immune response. The observed increase in serum TAC and catalase activity aligns with previous research Mahmoud et al. (2021), who reported a significant enhancement in antioxidant parameters of male Wistar rats fed NS seeds (30–50 g/kg) compared to a control diet. Further, Selim et al. (2019) demonstrated that dietary essential oils-isolated from NS improve blood oxidative stability, specifically by reducing MDA levels and enhancing catalase activity, which aligns with the results reported by Desai et al. (2015).

NS seeds are a rich source of antioxidants, notably phenolic and flavonoid compounds, which contribute significantly to their potent DPPH radical scavenging activity (Mahmoud et al. 2021). The link between DPPH scavenging activity and phenolic and flavonoid content is well-established, as documented by Adetuyi and Ibrahim (2014). This aligns with the observed antiviral properties of Nigella sativa (NS). Specifically, Ross (2023) identified alkaloids and flavonoids in NS that contribute to its anti-influenza activity. Beyond antiviral effects, Salem and Hossain (2000) emphasized NS's potential as a nutritional additive, citing its antioxidant and hepatoprotective properties.

The immune system is a vital defense mechanism that protects the body from various threats. It safeguards against pathogens, foreign invaders, and abnormal cells, thereby maintaining internal balance and overall health. It has been reported that adding 3% of NS to broiler diets significantly boosted the immune system (Khalaf et al. 2024). Immunological attributes such as IgG and IgM were significantly improved in Friesian calves given 10 g/kg starter (El-Nagar et al. 2023). These results are consistent with our data, as adding 3% or 1% of NS to starters promoted the production of IgG and IgM in preweaning calves. Several studies have indicated that NS supported the production of IgG and IgM in calves (Abd El-Hafeez et al. 2014; Fathi et al. 2024). Maintaining the immune system is crucial for sustaining the production cycle in dairy farms by reducing disease and mortality rates. *Salmonella* is a major cause of infectious diarrhea in ruminants. Studies have shown that NS-combined with nano-selenium exhibits antimicrobial activity against *Salmonella spp* (Abdalhamed et al. 2023). The antidiarrheal potential of NS in mice has recently been observed (Safitri et al. 2025), and may be linked to the presence of bioactive compounds such as flavonoids, alkaloids, tannins, and saponins.

Several studies have demonstrated the immunomodulatory effects of NS, which can be attributed to the presence of bioactive compounds like TQ. Key compounds in NS can activate the immune modulatory agent NF-κB pathway by upregulating phosphorylated P65 and I*κ*B*α*, as well as phosphorylating JNK, ERK, and p38 to activate the MAPK signaling pathway (Wei et al. 2022). These findings suggest that NS may serve as a potential dietary supplement to bolster immune function.

Inflammation is a natural and important bodily response to injury or infection. It's a complex process involving various immune cells and signaling molecules that work together to protect the body from harm. Our results indicated that All NS-supplemented groups showed a significant decrease in IL-4 (linear; *P* < 0.001) and a significant increase in IL-10 (linear; *P* < 0.001) levels in the plasma of pre-weaning calves. Interleukin-4 is a cytokine, a type of signaling molecule that plays a crucial role in the immune system of animals, including humans. It is primarily produced by T helper 2 (Th2) cells, a subset of white blood cells (Shankar et al. 2022). Several studies have shown that black seed and its active compounds, such as TQ, can modulate the immune system and reduce IL-4 production as shown in this study (Shahzad et al. 2009; Ciesielska-Figlon et al. 2023). The IL-10 is an anti-inflammatory cytokine, and black seed has been shown to have anti-inflammatory properties. Our study revealed that NS can promote IL-10 in calves. Black seeds can modulate the immune system, and this can include increasing the production of IL-10 (Hadi et al. 2016). Overall, the effect of black seed on IL-10 levels is complex and depends on various factors, including the specific condition, the dose and duration of treatment, and the individual's immune response. More research is needed to fully understand the mechanisms by which black seed affects IL-10 levels and the immune system. However, the available evidence suggests that black seeds may have potential as a natural remedy for certain inflammatory conditions. While this study provides valuable insights, it is important to acknowledge certain limitations. We focused solely on the effects of NS during the pre-weaning period, leaving the long-term effects unexplored. Additionally, due to resource constraints, we were unable to conduct further analyses, including gene expression, long term effects and intestinal microbiota assessments, which would have provided more comprehensive support for our conclusions.

## Conclusion

This research highlights the potential of 3% NS supplementation to optimize calf health and performance. We observed significant improvements in growth, feed efficiency, blood health, antioxidant levels, and immunity, coupled with a reduction in oxidative and inflammatory stress. These results suggest that incorporating NS into calf diets can bolster calf productivity and contribute to sustainable dairy farming practices by minimizing weaning-related challenges. While these results are promising, future molecular investigations are needed to strengthen our understanding of NS's mechanisms of action.

## Data Availability

All data included in this study are presented in the form of tables and figures.

## Abbreviations

*NS*: *Nigella sativa* seeds
*FBW*: Final body weight
*BWG*: Body weight gain
*TDMI*: Total dry matter intake
*FCR*: Feed conversion ratio
*TAC*: Total antioxidant capacity
*CAT*: Catalase
*IL-10*: Interleukin 10
*IL-4*: Interleukin 4
*MDA*: Malondialdehyde
*HGB*: Hemoglobin
*RBCs*: Red blood cell
*HCT*: Hematocrit
*MCHC*: Mean corpuscular hemoglobin concentration
*MCV*: Mean corpuscular volume
*PLAT*: Platelet
*MCH*: Mean corpuscular hemoglobin
*WBCs*: White Blood Cell
*LYM*: Lymphocytes
*MON*: Monocytes
*TC*: Total cholesterol
*AST*: Aspartate
*ALT*: Alanine transaminases
*TG*: Triglyceride
*HDL*: High-density lipoprotein
*VLDL*: Very low-density lipoprotein
*LDL*: Low-density lipoprotein
